# Electromagnetic induction heating for single crystal graphene growth: morphology control by rapid heating and quenching

**DOI:** 10.1038/srep09034

**Published:** 2015-03-12

**Authors:** Chaoxing Wu, Fushan Li, Wei Chen, Chandrasekar Perumal Veeramalai, Poh Choon Ooi, Tailiang Guo

**Affiliations:** Institute of Optoelectronic Technology, Fuzhou University, Fuzhou, 350002 People's Republic of China

**Keywords:** Synthesis of graphene, Two-dimensional materials

## Abstract

The direct observation of single crystal graphene growth and its shape evolution is of fundamental importance to the understanding of graphene growth physicochemical mechanisms and the achievement of wafer-scale single crystalline graphene. Here we demonstrate the controlled formation of single crystal graphene with varying shapes and directly observe the shape evolution of single crystal graphene by developing a localized-heating and rapid-quenching chemical vapor deposition (CVD) system based on electromagnetic induction heating. Importantly, rational control of circular, hexagonal and dendritic single crystalline graphene domains can be readily obtained for the first time by changing the growth condition. Systematic studies suggest that the graphene nucleation only occurs during the initial stage, while the domain density is independent of the growth temperatures due to the surface-limiting effect. In addition, the direct observation of graphene domain shape evolution is employed for the identification of competing growth mechanisms including diffusion-limited, attachment-limited and detachment-limited processes. Our study not only provides a novel method for morphology-controlled graphene synthesis, but also offers fundamental insights into the kinetics of single crystal graphene growth.

## Introduction

Chemical vapor deposition (CVD) has enabled the growth of single layer graphene over large area through the optimization of synthesis conditions^1,2,3^, including hydrogen and carbon precursors flow rates^4,5,6^, surface oxygen^7^, substrate^8,9^, temperature^10^ and pressure^11^. In order to achieve large size and high-quality single crystal graphene, a fundamental understanding of precise mechanisms that governs the formation of graphene domains is necessary. Much effort has been placed on the investigation of graphene growth kinetics by using carbon isotope labeling technique^7,12,13^, or the empirical parameters-controlled methods based on the nucleation and growth theory^14^. It has been widely accepted that the graphene growing process mainly involves surface catalytic reaction for catalyst with low-carbon solubility^1,13^. In this case, graphene nucleation on catalyst surface is one of the critical steps in the growth process. Various factors affect the initiation of graphene nucleation process, including the type or surface morphology of catalyst^15,16^, carbon source^17^, carbon segregation from metal-carbon melts^18^ and parameters in CVD growth^2,19,20^. In general, nucleation densities on polycrystal Cu substrates are nonuniform, representing a key problem in high quality graphene film synthesis. Recently it has been found that uniform nucleation distribution, low nucleation density and highly ordered single crystal graphene films can be obtained by using liquid Cu film as catalytic, which is possibly due to the elimination of Cu grain boundaries^21,22^. However, the direct insight of single crystal graphene growth and its corresponding domain shape evolution during the growth process are still lacking. The challenge is to suspend the graphene nucleation and growth process accurately so that one can observe the pristine graphene domain formation. But it is almost impossible to achieve this by using the conventional hot wall tube furnace due to the slow temperature ramping during the heating and cooling processes.

Electromagnetic induction heating is the process of heating an electrically conducting object by electromagnetic induction, where eddy currents are generated within the metal and resistance leads to Joule heating of the metal. Electromagnetic induction heating technique has been widely used in industry, including surface hardening, melting, brazing, sealing and heating to fit, due to its features such as fast-heating, fast-cooling, clean, cheap price and energy-saving. Although the technique has been demonstrated for the synthesis of carbon nanotubes^23,24^, zinc oxide nanowires and titanium dioxide nanoswords^25,26^, the great capacities of rapid heating and quenching characteristics belonging to electromagnetic induction heating have not been fully utilized. Here, for the first time we control the formation of varying shapes of single crystal graphene and directly observe the shape evolution by developing a localized-heating, rapid heating and quenching CVD system based on electromagnetic induction heating. The CVD system based on electromagnetic induction heating is capable of selectively heating the copper foil only and can also cool the copper foil from more than 1000°C to 700°C in ~5 s. By using the electromagnetic induction CVD system, we have identified the competing atomic phenomena, such as diffusion-limited growth, attachment-limited growth and detachment-limited growth, whose balancing effect defines the characteristic single crystal graphene domain density, shapes and crystal quality. Our study provides a novel method for morphology-controlled single crystal graphene synthesis and offers fundamental insights into the kinetics of growth processes.

## Results and Discussion

**Figure 1:**
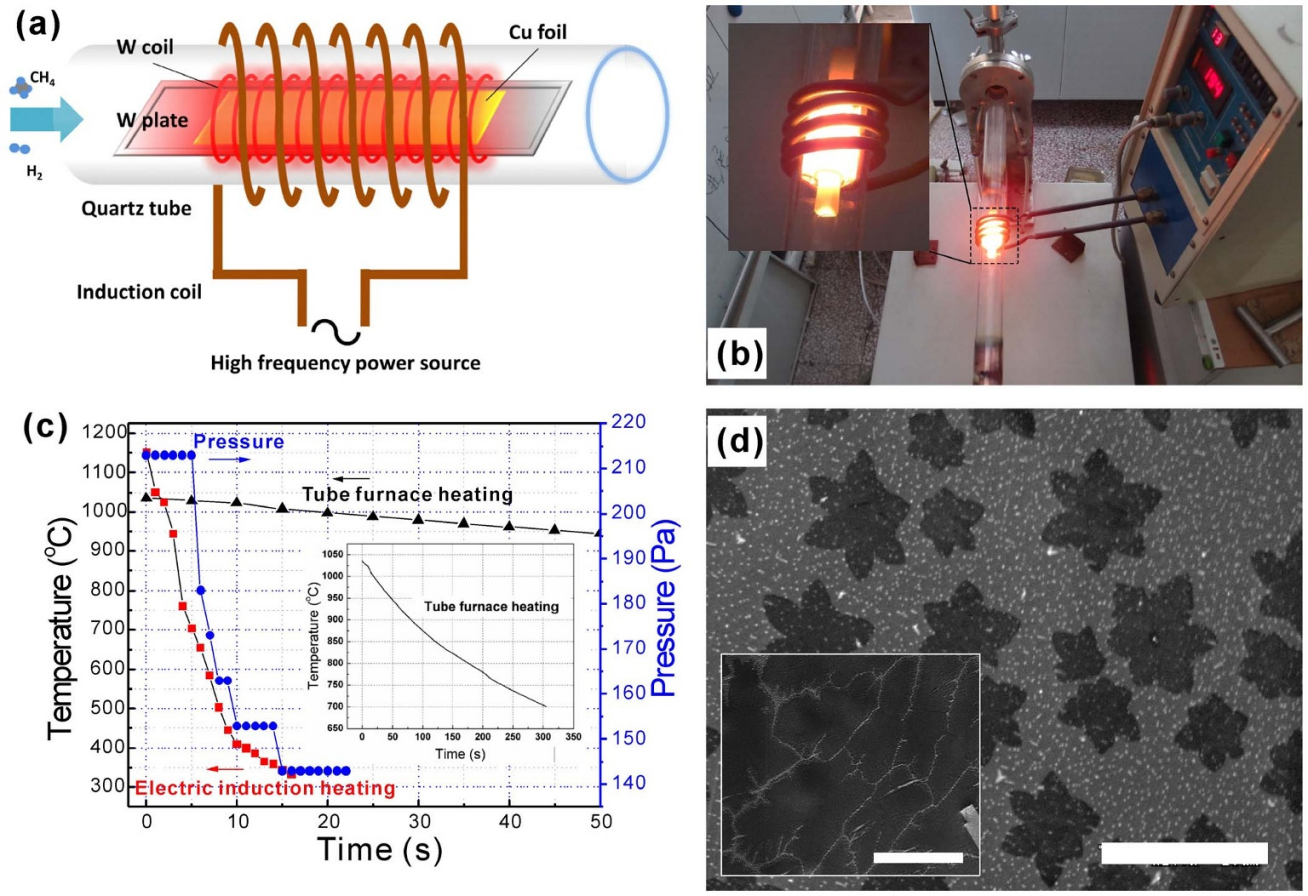
(a) Schematic structure of the CVD system. (b) Optical image of the CVD system, in which the selected W coil/W plate/Cu foils are heated. (c) Rapid-cooling performance of electromagnetic induction heating-based CVD system and its comparison with tube furnace-based system cooled by exposing to ambient environment (inset). The blue dot curve is the time-dependent chamber pressure variety when the CH_4_ source is cut off. (d) SEM image of graphene grown at 1035°C for 1 min by using electromagnetic induction heating and tube furnace heating (inset). Scale bar: 10 μm.

Figure 1a, b are the schematics and photographic pictures of the electromagnetic induction CVD system used in this work. A 2.5-cm-diam quartz tube, using as the CVD chamber, is wrapped by the Cu induction coil. The tungsten (W) coil/W plate/Cu foil package is then loaded into the quartz tube at the middle of Cu induction coil. When high frequency power is applied to the Cu induction coil, the W coil, W plate and the above Cu foil will be heated to a target temperature rapidly. However, the quartz tube chamber still keeps the low temperature. Thus the temperature of Cu foil will drop rapidly once the high frequency power is cut off. In general, there are two basic strategies for suspending the graphene growth process. The first method is to cut off the source of carbon such as CH_4_, which, however, requires more than 1 min for the carbon precursor gases to evacuate the chamber entirely due to the limited pump rate (Figure 1c). The alternate way is to cool down the reactor, which relies heavily on the cooling setup. The most important advantage of our electromagnetic induction CVD system is its localized-heating and rapid cooling capability. As shown in Figure 1c, for electromagnetic induction heating, it takes less than 5 s to decrease the temperature from 1050°C to 700°C, which is much less than that of tube furnace heating system (quenching the quartz tube in ambient environment). Thus the rapid cooling capability makes it possible to observe the time evolution of graphene domain formation and graphene growth and to contral graphene domains with various shapes at the beginning of nucleation. In order to further demonstrate the effect of cooling rate in the short-time graphene growth process, we investigated the formation of graphene at 1050°C for 1 min using electromagnetic induction CVD system and tube furnace CVD system, respectively. Compared to the separated star-liked graphene domain synthesize by electromagnetic induction heating method (Figure 1d), the domains synthesized by tube furnace are larger and almost fully cover the Cu substrate surface (inset of Figure 1d). Because in the temperature regime from target point (1050°C) to growth-stop point (~700°C), the as-synthesized graphene would grow larger. Thus it is impossible for us to correctly observe nucleation and growth in detail. On the contrary, nucleation step at the initial 5 s growth time could be observed by using the electromagnetic induction system.

**Figure 2:**
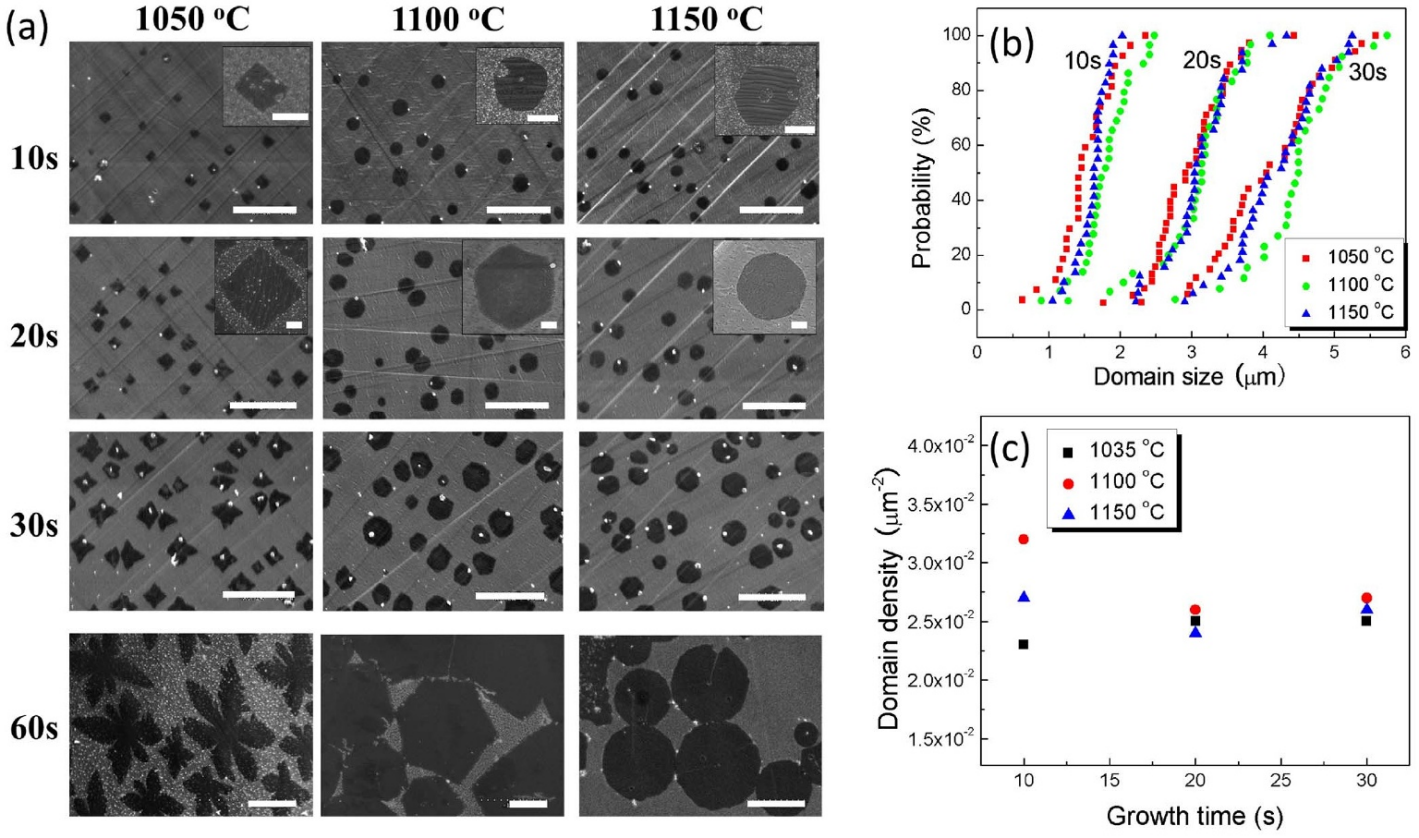
(a) SEM images of graphene domains grown on Cu at different growth temperatures and times. Scale bar: 10 μm; inset scale bar: 500 nm. Cumulative distribution plots of graphene domain size (b) and domain density distribution (c).

By employing the electromagnetic induction CVD system, the growth of single crystal graphene at 1050°C, 1100°C, 1150°C for duration ranging from 10 s to 60 s was investigated, as shown in the SEM images of Figure 2. The growth temperature range includes the maximum temperature (1150°C), which is above the Cu melting point (1083°C). Thus, the single crystal graphene is grown on the liquid Cu surface at 1100°C and 1150°C.

**Figure 3:**
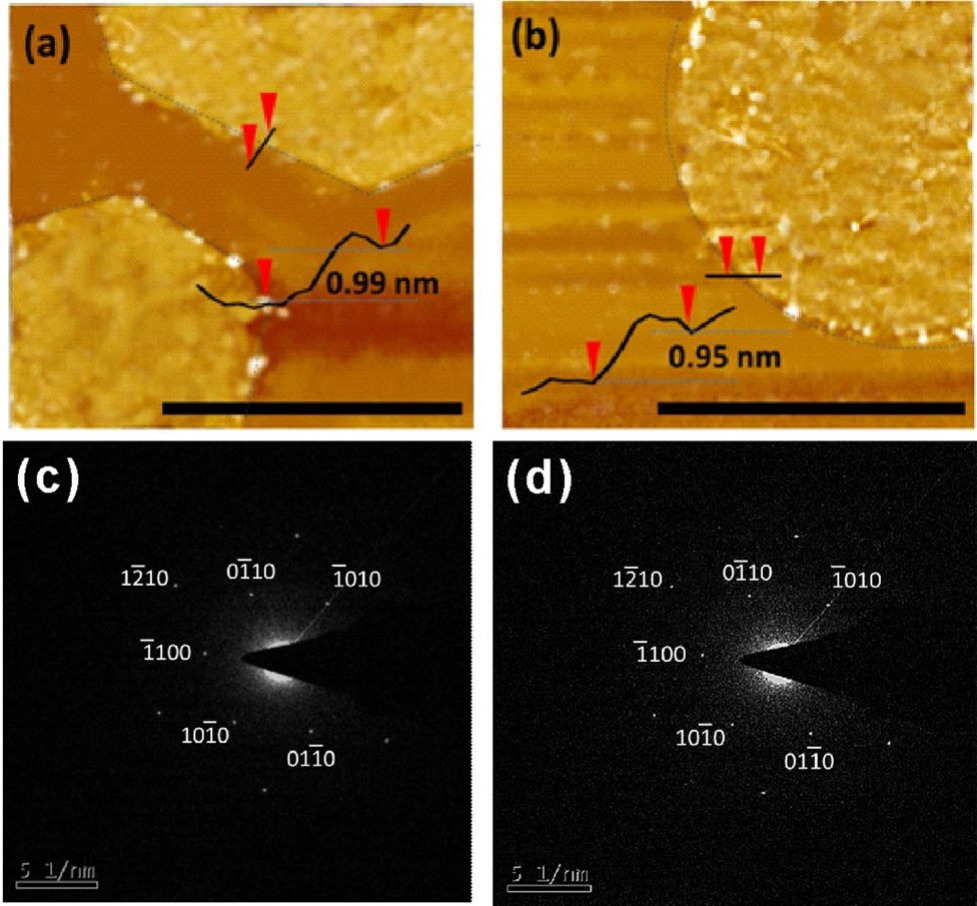
(a) AFM image of hexagonal graphene sheet and cross-sectional analysis. (b) AFM image of circular graphene sheet and cross-sectional analysis. Scale bar: 1 μm. SAED patterns taken from (c) hexagonal graphene and (d) circular graphene.

The thickness of the as-synthesized graphene sheets transferred onto Si substrate was further measured by atomic force microscope (AFM), as shown in Figure 3. Figure 3a and Figure 3b present the AFM images of hexagonal and circular graphene sheets, respectively. The inset line scan profiles show that the thickness of the graphene sheets was smaller than 1 nm. The results indicate that the single layer graphene sheets were obtained in our experiments. To confirm the crystallinity of the circular graphene domain, we have conducted selective area electron diffraction (SAED) on the circular graphene domain. As shown in Figure 3c and Figure 3d, the circular and the hexagonal graphene domain shows the same crystalline structure.

By using this ultra rapid cooling method, we have observed some remarkable key phenomena as follows: (1) the graphene domain density and lateral size are independent of growth temperatures (Figure 2b); (2) nucleation only occurs during the initial instants and almost no new nuclei are formed with increase of growth time (Figure 2c);(3) variable shapes of graphene domains, e.g., circular, hexagonal and dendritic, are under the control of growth temperatures; (4) the temperature would affect the shape evolution of graphene domains.

**Figure 4:**
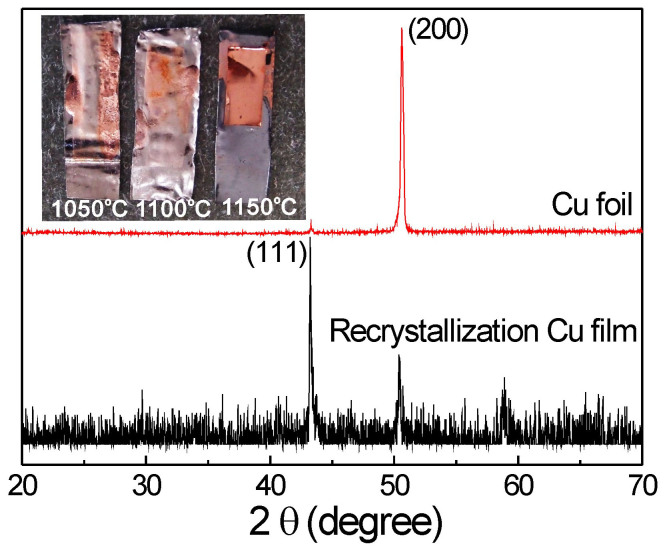
XRD patterns of annealed Cu foil and recrystallized Cu film. Inset is the optical photos of Cu catalyst after graphene synthesis and the temperatures are 1050°C, 1100°C and 1150°C, respectively.

The density of graphene domains is independent of the growth temperature under our experimental conditions within the growth temperatures range from 1050°C to 1150°C, which is in contrast to the reported literatures^14,27^. According to the Robinson and Robins model^28^, the nucleation is the result of competition between carbon species adsorption, surface diffusion and re-evaporation processes^29^. One of the possible reasons for the temperature-independent nucleation density is that the catalyst used in our study is liquid phase Cu film (at 1100°C and 1150°C) and recrystallized Cu film (at 1050°C). Figure 4 shows the XRD patterns and optical photos of Cu foil (after 1050°C reaction) and recrystallized Cu film. The Cu (111) surface can be obtained with recrystallized Cu foil. According to the simulation studies, C_2_H_2_ can be easily formed from CH_4_ on the Cu (111) surface^30^. Therefore, the Cu (111) surface represents a more favorable reaction pathway for nucleation. Moreover, the diffusion of carbon species groups is much faster on (111) surface than that on (200) surface of commonly used polycrystalline Cu foil^30^. We believe that the nucleation kinetics is depended mostly on the surface state of Cu catalyst rather than on the growth temperature. It is proposed that the temperature-independent nucleation density on recrystallized Cu film is the result of formation of Cu (111) surface. Even though other peaks except (111) also appear for recrystallized Cu film, the (111) peak is sharp and stronger than other peaks. Thus for recrystallized Cu film, the Cu (111) surface is dominant. Furthermore, the carbon species can easily form and nucleate on the dominant Cu (111) surface, which may retrain the graphene nucleation on other surface. Although there is lack of theoretical study explaining the critical role of liquid Cu surface playing in graphene nucleation, the similar nucleation behavior on solid Cu (111) surface and liquid Cu surface demonstrates that the atomic phenomena of carbon species on liquid Cu surface is the same to that on the Cu (111) surface.

**Figure 5:**
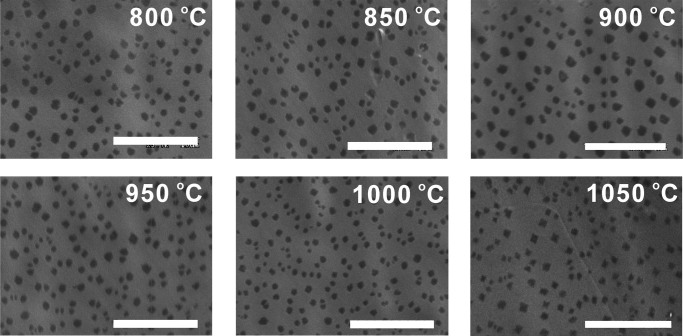
SEM images of graphene domains grown on recrystallized Cu film for 10 s at different temperatures. Scale bar: 10 μm.

As reported, the graphene nuclei density decreases with the increase of growth temperature^14,27^. It is believed that the graphene nuclei will keep on growing or even connect with each other during the conventional slow cooling process (last for several minutes). Particularly, when the growth temperature increases, the domain aggregation will become notable, which leads to the seeming result that domain density is decreased with increase of temperature. With our rapid-quenching CVD system, we are able to rule out the interference of domain aggregation on the graphene domain density observation. We investigated the growth of graphene on the recrystallized Cu film from 800°C to 1050°C for 10 s. As shown in the SEM images of Figure 5, the initial graphene domain density shows unobvious dependence on the growth temperature ranged from 800°C to 1050°C. According to the growth mechanism of graphene formation on Cu surface, upon the breakdown of methane on the Cu surface, the concentration of the active carbon species increases to a critical super saturation level, where the nucleation of stable graphene nuclei takes place. As previously reported^31^, the carbon-Cu interaction is sufficiently weak, the decomposed carbon atoms are only able to diffuse on the Cu surface and both the nucleation and growth of graphene are dominated by the surface diffusion of the decomposed carbon atoms. As a consequence, the feedstock cannot be accessed for the catalyst surface covered with carbon species, namely self-surface-limited effect. Therefore, the surface carbon species supersaturation concentration is limited mostly by the Cu surface rather than by the growth temperature, which leads to the temperature-independent nucleation density.

Unlike the domain density, the shape of graphene domains, for instance, circular, hexagonal and dendritic, is under the control of growth temperatures. With the increase of growth temperature, the single crystal graphene domains are transformed from dendritic shape (at 1050°C) to hexagonal shape (at 1100°C) and to circular shape (at 1150°C). Although the graphene domains with dendritic and hexagonal shape have been previously synthesized on Cu foil or liquid Cu film^19,21,22^, the circular shape graphene domains are rarely reported. It is worth noting that circular shape graphene domains can only be synthesized at high temperatures (above 1150°C). However, due to the relatively low heating and cooling rate of traditional tube furnace, the complete evaporation of Cu substrate would occur during the heating, growing and cooling processes. That is the reason why it is hard to observe circular shape graphene domains by the conventional CVD synthesis methods. The graphene domains are dendritic shape after 60 s growth at 1050°C. For the graphene domains grown at 1100°C and 1150°C for 60 s, the size keeps the same order of magnitudes, but the shape is transformed from dendritic to hexagon and circular, respectively. The time-evolution of domain growth at different growth temperatures could be visualized by rapid cooling the graphene growth process. The graphene domains grown at 1050°C evolve from diamond to tetragonal-star and then hexangular star shape. The domains grown at 1100°C remain hexagon, while the domains grown at 1150°C evolve from quasi-hexagonal to circular shape as they grew. According to the second law of thermodynamics, in the chemical reaction process, including CVD, a chemical reaction will proceed if the change in Gibbs free energy is negative. In other word, the thermodynamic driving force for a chemical reaction is the negative change of Gibbs free energy. In our graphene CVD synthesis process, the carbon atom in a state of gaseous species changed into that in the solid graphene state through the chemical reaction and the graphene domains were growing larger under the suitable condition. Thus the total Gibbs free energy is reduced, which is the thermodynamic driving force to grow graphene. So we try to explain the formation of different graphene domain shapes from the perspective of thermodynamics.

**Figure 6:**
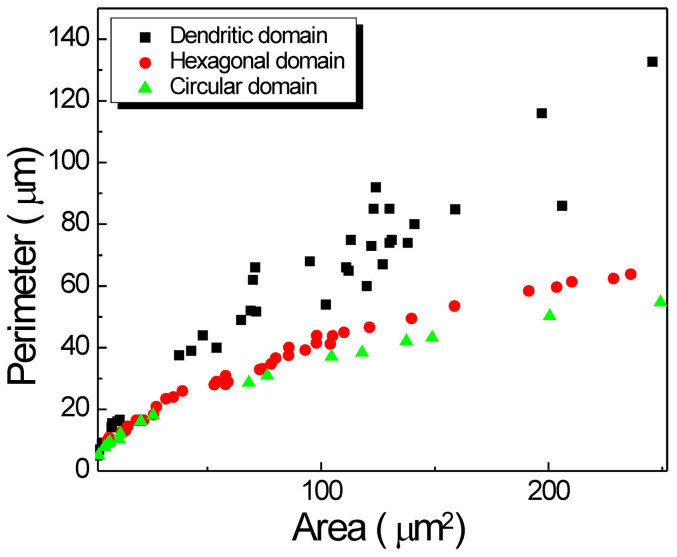
Perimeter-area relation of single crystal graphene domains grown at different growth temperatures and times. The distribution was obtained by plotting the perimeter-area of circular, hexagonal and dendritic graphene single crystals, respectively. The single crystals with different size, which was achieved by changing the growth time, were taken into count.

According to the Gibbs thermodynamic principle, the shape of an equilibrium crystal is obtained by minimizing the total surface free energy associated to the crystal-medium interface^32^. The change of Gibbs free energy induced by the formation of graphene from gaseous CH_4_ can be expressed as:

Where *A* is the area of graphene domain, *A*_*o*_ is the equivalent area of single carbon atom in graphene, *Δg* is the change of Gibbs free energy from single gaseous CH_4_ molecule to solid graphene lattice, *k* is the shape factor, which reflects the anisotropy of graphene, *C*(*A*) is the perimeter of graphene domain, which is sensitively dependent on the shape of graphene domain, γ_*edge*_ is the edge free energy of graphene domain, *A*_*Cuf*_ is the area of Cu-CH_4_:H_2_ flow surface, γ_*Gf*_, γ_*GCu*_, γ_*Cuf*_ is the interfacial energy of grapnene-CH_4_:H_2_ flow surface, graphene-Cu surface and Cu-CH_4_:H_2_ flow surface, respectively. The first term reflects the final chemical energy variation tendency during the growth process, which is the thermodynamic driving force of graphene growth. The second and third terms reflect the thermodynamic impediment of graphene growth by the introduction of additional edge energy and interfacial energy of the total CVD system. The final Gibbs free energy of the CVD system is tending to reach the minimal value as the result of the competition among the three parts in the equation (1). The growth conditions will determine which part plays as the critical role and will affect the shape of the graphene domains. Note that the size of graphene domains grown at 1050°C, 1100°C and 1150°C keeps the same order of magnitudes. Thus the effect of interfacial energy on the varying shapes can be ruled out. When the areas of the two-terminal domains are the same, the perimeter depends on the shape of the domain sensitively. Figure 6 shows the experimental relationship between perimeter and area of the graphene domains at different growth temperatures. It can be seen that the circular graphene domain has the smallest perimeter, while the dendritic shape graphene domain has the largest perimeter. When the areas of the two-terminal domains are the same, the edge energy is the only difference, depending on the perimeter of domain sensitively. Thus the graphene domain grown at 1150°C has the lowest Gibbs free energy. Although the graphene domain grown at 1050°C has relatively higher Gibbs free energy, its dendritic edges make it possible to reduce the total energy of CVD system at 1050°C, which will be discussed below.

The overall processes of CH_4_ on Cu surface during the graphene growth are: (1) active CH_4_ is broke down into carbon species such as CH_x_ (x = 0–3) through dissociative chemisorption on the Cu surface and diffuse on the Cu surface; (2) once the increasing dynamic concentration of carbon species reaches critical supersaturation level, the nucleation of graphene takes place; (3) part of the supersaturated carbon species with enough energy reach the graphene domain edge and attach to the graphene domain; (4) the CH_x_ species at the unstable graphene edge detached itself from graphene and form dissociative carbon species; (5) Dissociative carbon species combine with hydrogen and are desorbed from Cu surface^7,30^.

**Figure 7:**
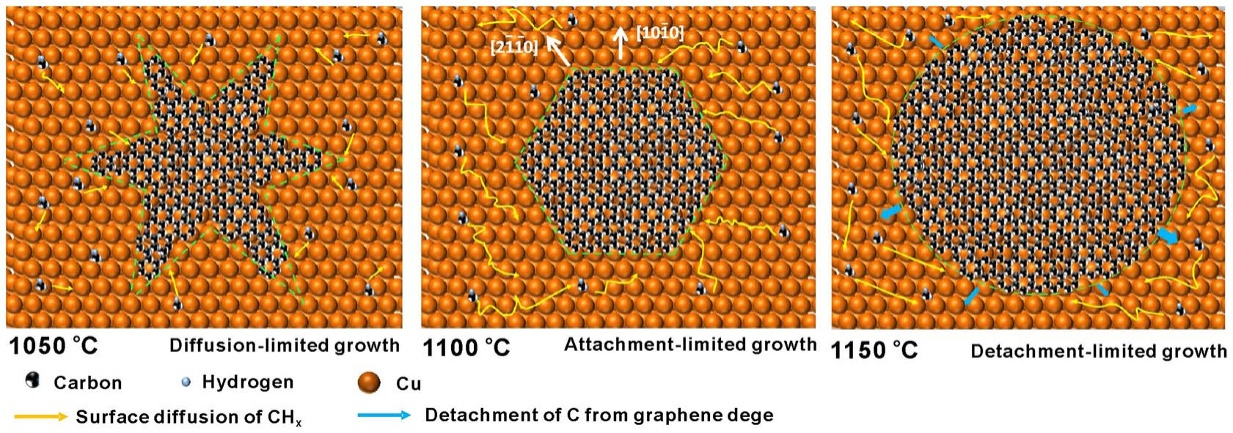
Atomic-scale schematics of graphene domains growth on Cu at different temperature.

Figure 7 shows the atomic-scale schematics of graphene growth on Cu at different temperatures. The typical shape of graphene domains, hexagons with sharp edges, is obtained from the kinetic Wulff construction^32^. It is expected that the growth limiting factor for hexagon graphene domain is the attachment of carbon species at the anisotropic growing front of natural graphene nucleus. The dissociative carbon species on the liquid Cu surface (at 1100°C) have enough concentration and high diffusing ability. Because the natural graphene domain exhibits fast growth in the [2īī0] direction and slow growth in the [10ī0] direction, the typical hexagons domain is formed as it growth. As the temperature decreases to 1050°C, the surface diffusing ability of carbon species on solid Cu surface is limited, thus the dendritic graphene is under the control of diffusion-limited growth mechanism^33^. It should be noted that the dendritic shape has the longest perimeter in comparison with other shapes, which is beneficial for the collection of dissociative carbon species. Moreover, the dendritic shape becomes manifest as the domain grows. The achievement of carbon species attachment to domain edge and incorporation into graphene lattice before their desorption can lower the total Gibbs energy of the CVD system, which is the thermodynamic driving force for the dendritic graphene domain formation. The formation of circular graphene indicates that, with the increase of temperature, attachment and surface diffusion are no longer the limiting factors. As mentioned above, the circular graphene domain grown at 1150°C has the lowest Gibbs energy due to the shortest edge. Although the zigzag edge along [10ī0] direction has the lower edge free energy than that of armchair edge, its longer perimeter will increase the total Gibbs energy of hexagons domain especially when the domain grows larger. Thus the anisotropic edge energy is no longer the limiting factor and the shape is instead governed by the minimization of perimeter. It is supposed that the high temperature makes the active CH_x_ at the hexagonal graphene edge detach from graphene (detachment-limited growth) and relax to the kink with lower energy, which lead to the circular shape on the macro level. The phenomenon of shape evolution of 2-dimensional crystal has also been proved theoretically^34^.

## Conclusions

In conclusion, we developed a rapid-quenching CVD system based on electromagnetic induction heating, which is capable of selectively heating the Cu substrate only and rapid cooling the Cu substrate from 1150°C to 700°C in several seconds. The rapid-quenching CVD system makes it possible to terminate the graphene growth rapidly and to study the graphene growth kinetics. Based on the rapid-quenching CVD system, our observation shows that the graphene nucleation only occurs during the initial stage and the domain density is independent of the growth temperatures due to the surface-limiting effect. Taking full advantage of the rapid-heating and rapid-cooling method, controllable circular, hexagonal and dendritic graphene domains were obtained. The evolution of varying shapes with growth time were observed, which are beneficial for the deep identification of competing atomic phenomena, such as diffusion-limited, attachment-limited and detachment-limited mechanism, in single crystal graphene growth.

## Methods

### Synthesis of graphene

Graphene was synthesized by electromagnetic induction heating CVD using a gas mixture of CH_4_ and H_2_, in which CH_4_ was used as the carbon-containing precursor. A 2.5-cm-diam quartz tube, using as the CVD chamber, is wrapped by the Cu induction coil. The Cu foil (1 cm × 2 cm, 25-μm-thick, 99.8%, Alfa Aesar, 13382) using as graphene growth catalyst was placed on a tungsten (W) plate (1.2 cm × 4 cm), which was put into a W coil (with inner diameter of 1.2 cm and 3-cm-length). The package was then loaded into the quartz tube at the middle of Cu induction coil. When the high frequency power (home-built electromagnetic induction heating machine, GY-25A, 60 kHz) was applied on the Cu induction coil, the Cu foil was heated to a high temperature rapidly. The temperature was measured by using a thermocouple (model: XMT-101, accuracy class: 1.0). Before the growth of graphene, the Cu foils on W plate were firstly annealed at 1150°C for 10 s. During the growing process, 28 sccm H_2_ and 19 sccm CH_4_ were introduced to the CVD chamber at pressure of 215 Pa and the high power was applied to the Cu foil to rise up to target temperature. After reaction, the Cu foil was cooled down rapidly with the flow of 28 sccm H_2_.

### Characterizations

Scanning electron microscopy (SEM) images of graphene on Cu foil were obtained with Hitachi S-3000N and Nova NanoSEM 230 at 5 kV. High resolution transmission electron microscopy (HRTEM) selected area electron diffraction (SAED) patterns were taken at 80 kV with TECNAI G2F20. Atomic force microscopy (AFM) images of graphene on silicon substrate were obtained with Bruker Multimode 8 at tapping mode. X-Ray Diffraction (XRD) patterns of the Cu foil and the recrystallized Cu film were obtained with MiniFlex II.
